# Website Analytics of a Google Ads Campaign for a Men’s Mental Health Website: Comparative Analysis

**DOI:** 10.2196/12428

**Published:** 2018-12-13

**Authors:** Andrea Lynn Murphy, Sophie Peltekian, David M Gardner

**Affiliations:** 1 College of Pharmacy Faculty of Health Dalhousie University Halifax, NS Canada; 2 Department of Psychiatry Faculty of Medicine Dalhousie University Halifax, NS Canada

**Keywords:** alcohol, alcoholism, analytics, anxiety, consumer health informatics, depression, Google Ads, insomnia, men, suicide, tobacco, tobacco use

## Abstract

**Background:**

Men with mental health and addictions problems seek information and help from health service providers and community support less often than women with such problems. Online health resources offer men rapid access to self-care recommendations and resources and anonymity; however, only a few websites are specifically developed for men. Headstrong - Taking Things Head-On was a community pharmacy and online health promotion initiative for men living with mental health and addictions problems. The Headstrong website was developed to offer a curated collection of print and online recommended resources (primarily self-help oriented) for depression, anxiety, insomnia, tobacco and alcohol use problems, and suicide. To increase awareness of the initiative and use of the website’s content and resource recommendations, a Google Ads campaign was developed.

**Objective:**

This study aimed to compare user acquisition and behavior on the Headstrong website during and after a Google Ads campaign.

**Methods:**

The Google Ads campaign was launched on December 21, 2017, and run until February 28, 2018. Website analytics (acquisition of new users, behavior in terms of at-website actions and duration, devices used, and conversions [link-outs to recommended resources]) in a 30-day period during the campaign (January 26, 2018 to February 24, 2018) were compared to a similar 30-day period after the campaign (March 23, 2018 to April 21, 2018). A cost analysis of the ad campaign was also performed.

**Results:**

The ad campaign generated 3011 clicks and 4.5 million impressions in total. In addition, the campaign received 1311 website users during the 30-day period of the ad campaign as compared to 241 users during the 30-day period after the ad campaign (*P*<.001). Return visitor (17.7% vs 27.8%) and nonbounce (19.5% vs 39.8%) user rates as well as session duration (42 vs 102 seconds) and page views per session (1.4 vs 2.1) were lower during the ad campaign than after the campaign (*P*<.01 for all). The 30-day period of the ad campaign included 9 sessions with conversions initiated by an ad click. Paid and display ads accounted for 63% of the site traffic during the ad campaign, most of which came from mobile phone users. Desktops were the most-common device used after the ad campaign acquired the website via direct and organic searches primarily (92%). The estimated cost per session with one or more conversions was Can $54.69 and cost per conversion was Can $32.81.

**Conclusions:**

A Google Ads campaign designed to direct men to the Headstrong website increased the number of user visits by more than five-fold. However, engagement by users responding to the ad campaign was substantially lower than that by users who visited the website via other acquisition methods, possibly reflecting the nonspecific online targeting of men by the ad campaign. General targeting of men online to promote men’s mental health appears to have limited value.

Original Paper

## Introduction

“Headstrong - Taking Things Head On,” hereafter referred to as the Headstrong initiative and website [1], targeted men living with mental health and addictions problems in Nova Scotia, Canada. The use of male-specific interventions is a part of current recommendations to engage men in their mental health [2]. The internet has become a major source for users to acquire health information [3]. Recently, internet-based interventions in a mental health context were found to show beneficial effects [4]. Additionally, some reports suggest that men may be more likely to independently seek electronic health information than consult a health care professional [5-8].

Similar to other mental health promotion websites for men, the Headstrong website aimed to provide a male-friendly medium to start the self-help-seeking process [9]. The Headstrong website was accessible to men searching the internet on their own or when used in conjunction with other resources and was based on referral from a community pharmacist participating in the Headstrong initiative [1]. The main components of the Headstrong initiative included providing pharmacists with education, training, and resources including the Headstrong website to help promote men’s mental health. The objectives of the initiative were to promote access to resources available to the public through community pharmacies and the Headstrong website and to provide pharmacists with a process, knowledge, and resources to help men. The Headstrong website was developed and designed, with feedback from the project’s “male mentors,” specifically to engage men in order to help them identify opportunities to address selected mental health and substance use issues. It provided a curated library of recommended print and electronic resources for self-help on depression; anxiety; insomnia; problems with alcohol and tobacco use; and thoughts, intentions, and behaviors related to suicide.

The initiative and website were promoted around the project launch in October 2017 through in-pharmacy advertising and existing relationships with pharmacists and clients. In addition, the initiative and website were promoted through social media (Twitter and Facebook) and word of mouth. To increase awareness of the initiative and website, a Google Ads campaign (formerly, Google Adwords [10]) was developed to attract men living in Nova Scotia who searched for Headstrong website topics and performed general internet searches. Google Ads have been used in health-related contexts such as for patient recruitment in studies [11-14], web-based interventions [15-18], and increasing awareness of health promotion campaigns [19-23], as was the case with Headstrong website. There have been mixed results regarding the success of Google Ads campaigns compared to other digital advertising mediums such as Facebook advertisements [12,15,17].

**Figure 1:**
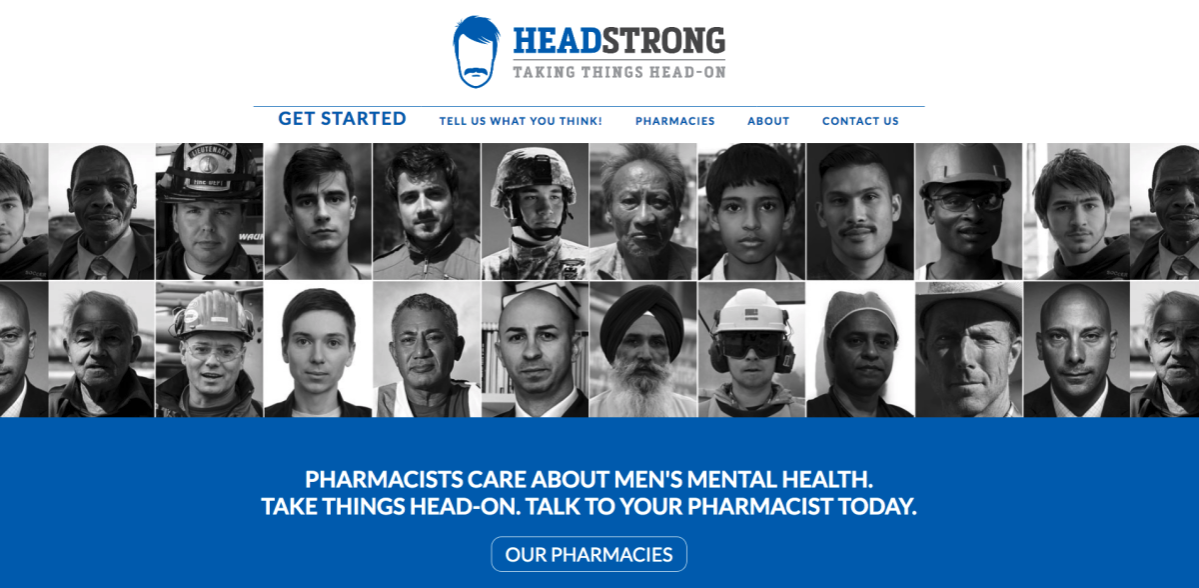
Headstrong.life homepage.

The primary objective of this study was to compare user behavior during and after a Google Ads campaign on the Headstrong website (Figure 1) and analyze the associated cost.

## Methods

### Design

**Figure 2:**
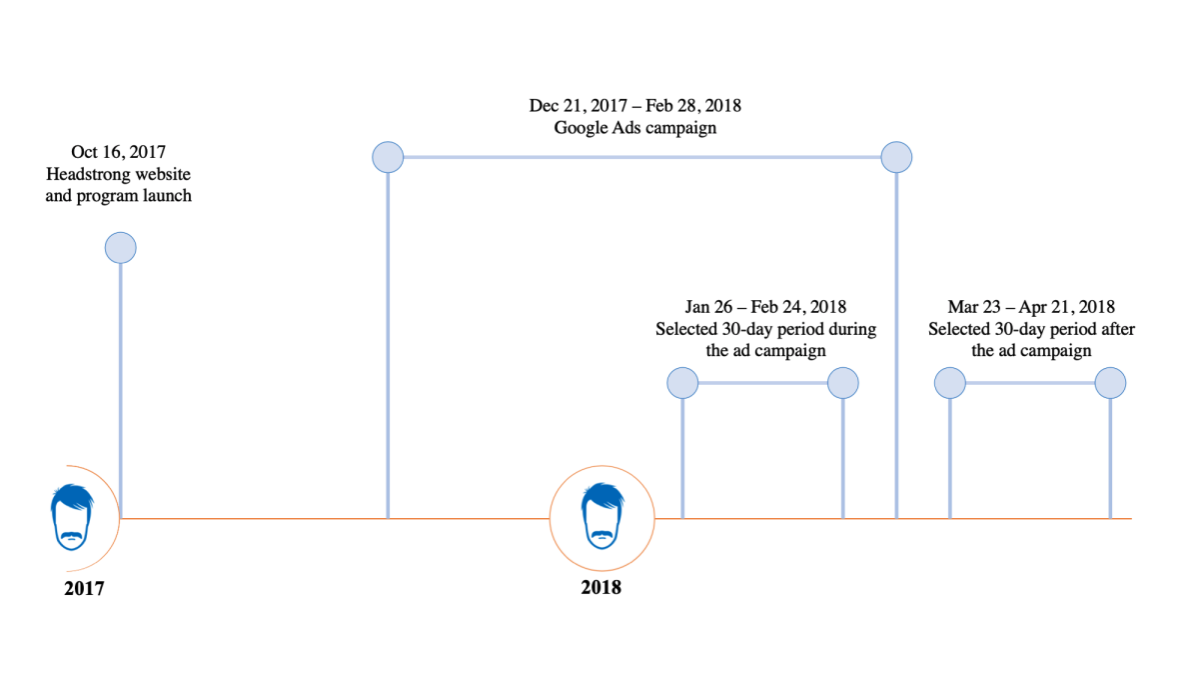
Timeline of the Headstrong – Taking Things Head-On initiative and the Google Ads campaign.

This study examined website analytic measures for the Headstrong website during and after the Google Ads campaign. The Headstrong website and initiative were launched on October 16, 2017. The Google Ads campaign was launched on December 21, 2017, and run until February 28, 2018. A 30-day period during the campaign (January 26, 2018 to February 24, 2018) was compared to a similar 30-day period after the campaign (March 23, 2018 to April 21, 2018). For the ad campaign period, we chose a 30-day range that did not include religious holidays, work or school vacations, or a broadcast call-in radio show that featured the website and profoundly influenced user traffic. Additionally, we changed the daily budget for our ad campaign from $30 to $15 per day. After observing an increase in traffic as a result of the ad campaign, we reduced the daily budget to extend the ad campaign for a longer period of time. The selected 30-day ad campaign range was during the $15 per day period. The 30-day period selected after the ad campaign coincided with the end of the Headstrong initiative in pharmacies (Figure 2).

Headstrong website visits were observed over the course of the Headstrong initiative, and website analytics were compared during and after the campaign for website acquisition. In addition, the devices used to access the Headstrong website and geographical targeting of Nova Scotia users were analyzed. Further, a cost analysis of the Google Ads campaign and specific ad groups were assessed. Google analytics terms used throughout this study are defined in Table 1.

Using a cost-per-click model, the Headstrong Google Ads campaign was geographically limited to users living in the province of Nova Scotia. The campaign featured numerous keywords to ensure that a broad range of people living in Nova Scotia could find the website in their search efforts. Specific keywords, supported by direct consultation with Google, were selected to help people already searching for mental health information, resources, or support in Nova Scotia to learn of the Headstrong website and its recommended resources. Nonspecific key words were also included to raise awareness of the website among men who were online for other reasons. A sample of keywords used in the campaign are provided in Table 2.

**Figure 3:**
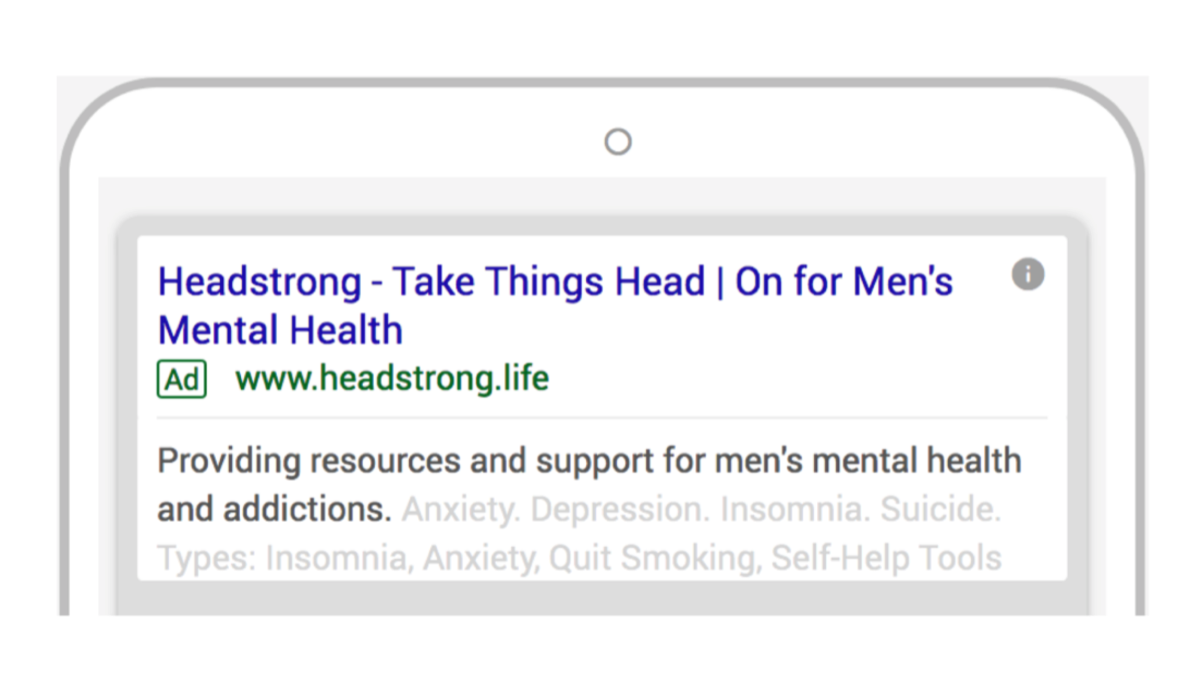
Sample advertisement from the Google ads campaign of the Headstrong website for mobile devices.

The campaign type used was “Search Network with Display,” which allows the advertisement to appear in Google search results for key terms outlined by the Headstrong team and on various websites, selected by Google, that were expected to be of interest to potential users based on their online activity [24]. Use of the display ads in addition to the Search Network option increased the opportunity to reach a wide audience. A sample of an advertisement that appeared for mobile devices is shown in Figure 3.

### Statistical Analysis

Descriptive statistics were used to characterize user behavior. The Poisson mean test was performed to compare the rate of daily use of the website during and after the ad campaign. We used the Fisher exact test and Chi-square analysis for dichotomous data, and the unpaired *t*-test for analysis of continuous variables. Variances are expressed as standard deviations. Cost data are reported in Canadian dollars unless stated otherwise.

**Table 1:** Definitions of Google analytics terminology.

Terminology		Explanation	
**Analytics**			
	Average pages per session		The average number of pages viewed during a session on the website
	Average session duration		Total duration of all sessions (in seconds) divided by the number of sessions
	Bounce rate		The percentage of sessions that a person leaves the website from the landing page without browsing any further
	Click-through rate		The percentage of users who view the ad and then click the ad
	Conversions		A desired action once a user interacts with the ad. For the Headstrong website, clicking on recommended resources counted as a conversion
	Conversion rate		The percentage of sessions on the website that lead to clicking of an outbound resource link
	Cost per click		The amount paid to the advertiser each time an ad is clicked
	Impressions		How many times the ad is viewed in any form on the internet
	Nonbounce user		A user who proceeds to interact with the website after arriving on the landing page
	Returning user		When the same user has more than one session
	Session		A set of user interactions with the website that take place within a given time frame; a single session can contain multiple page views
**Acquisition**			
	Direct		Users who visited Headstrong by typing the website directly into their internet browser
	Display ad		Paid advertisements that appeared on the side of the user’s internet browser while browsing the internet on various websites determined by Google Ads for their relevance and suitability
	Organic		Users who searched for the Headstrong website directly through a search engine
	Paid search		Users who were specifically searching key terms of the Headstrong website Google Ads campaign
	Referral		Users who found the website through some other website (not paid advertising)
	Social		Users who found the website through a social media channel

**Table 2:** Samples of keyword search terms used in the Headstrong Google Ads campaign.

Ad group	Keywords^a^
Depression	Depression, “Depression”, Clinical Depression, “Depression symptoms”, Depression and Health, “Major Depression”, Depression support
Mental health	“Mental health”, “Mental Issues”, Mental health info, Mental wellness, “Mental Health Helpline”, Mental information, “Mental health is”
Smoking	“Quit smoking”, “Smoking cessation”, “Smoking”
General keywords: No-ad group	“Stress”, “Drinking”, “Insomnia”, “Pharmacist”, “Anxiety”, “Anger”, “Alcohol”, “Self harm”, “CBT”, “Nova Scotia”

^a^Keywords in quotation marks are referred to as “phrases”; these search terms were used exactly as seen in the quotation marks to present an advertisement to the potential user. Keywords without quotation marks can signal an advertisement for a potential user whose search includes the word, regardless of other words included in their search.

## Results

### Overall Campaign

**Figure 4:**
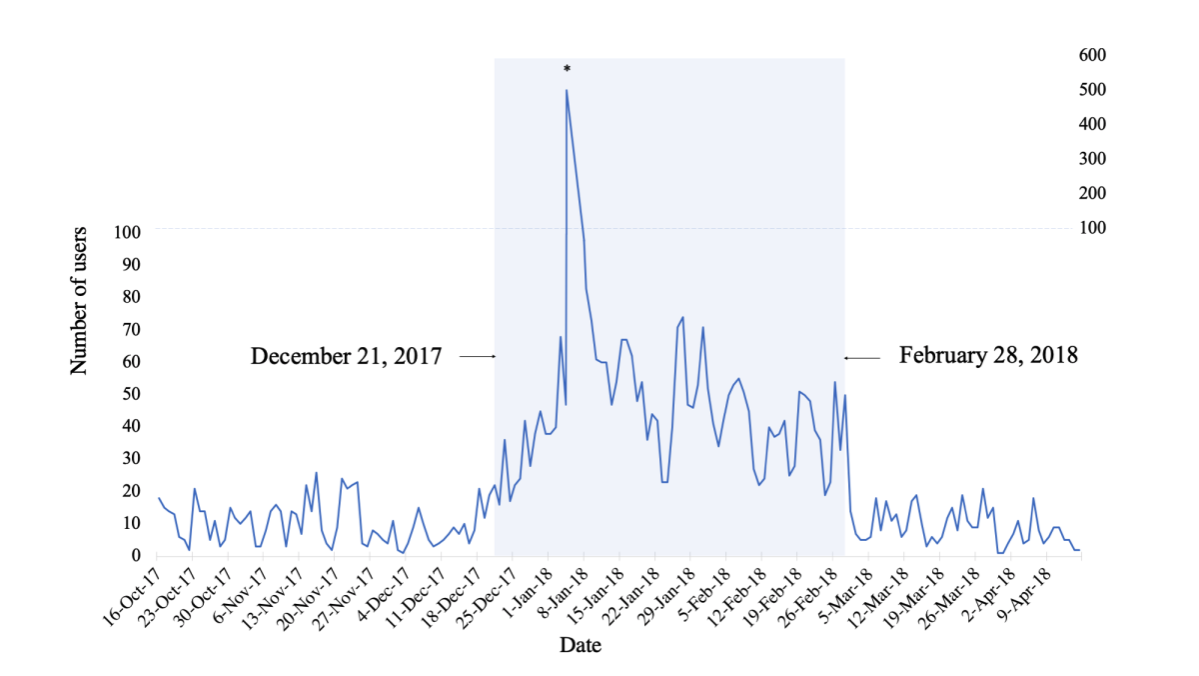
Daily rate of Headstrong website visits. *On January 5, 2018, an Ontario-wide call-in public radio show about insomnia promoted resources available on the Headstrong website, which resulted in a high volume of visits for several days thereafter.

The 69-day Google Ads campaign was initiated 66 days after the launch of the Headstrong initiative and resulted in 3011 visits to the Headstrong website and 4.5 million impressions. Daily counts of site visits from the launch of the website (October 16, 2017) to the end of the analysis period (April 25, 2018) are shown in Figure 4. The rate of visits was higher during the ad campaign (December 21, 2017 to February 28, 2018) than before or after the campaign.

### Comparative Analyses: During and After the Ad Campaign

#### Analytics

**Figure 5:**
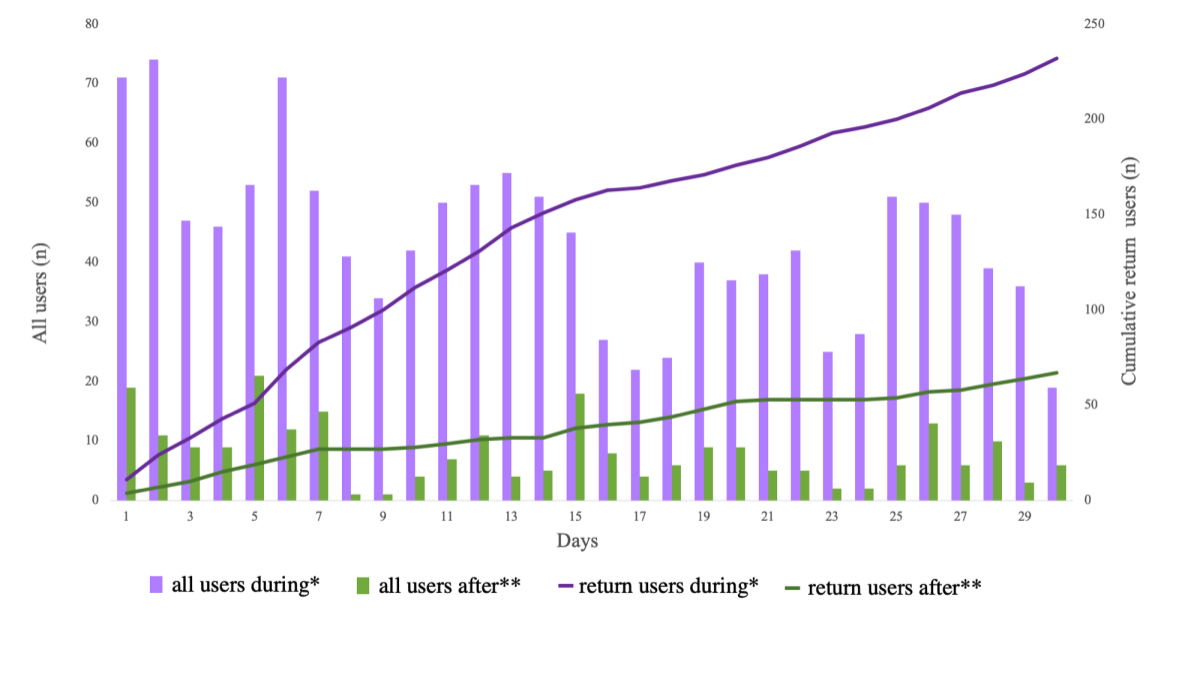
All website visits by all users and return users during and after the ad campaign.*The 30-day period during the ad campaign (January 26, 2018 to February 24, 2018). **The 30-day period after the ad campaign (March 23, 2018 to April 21, 2018).

**Figure 6:**
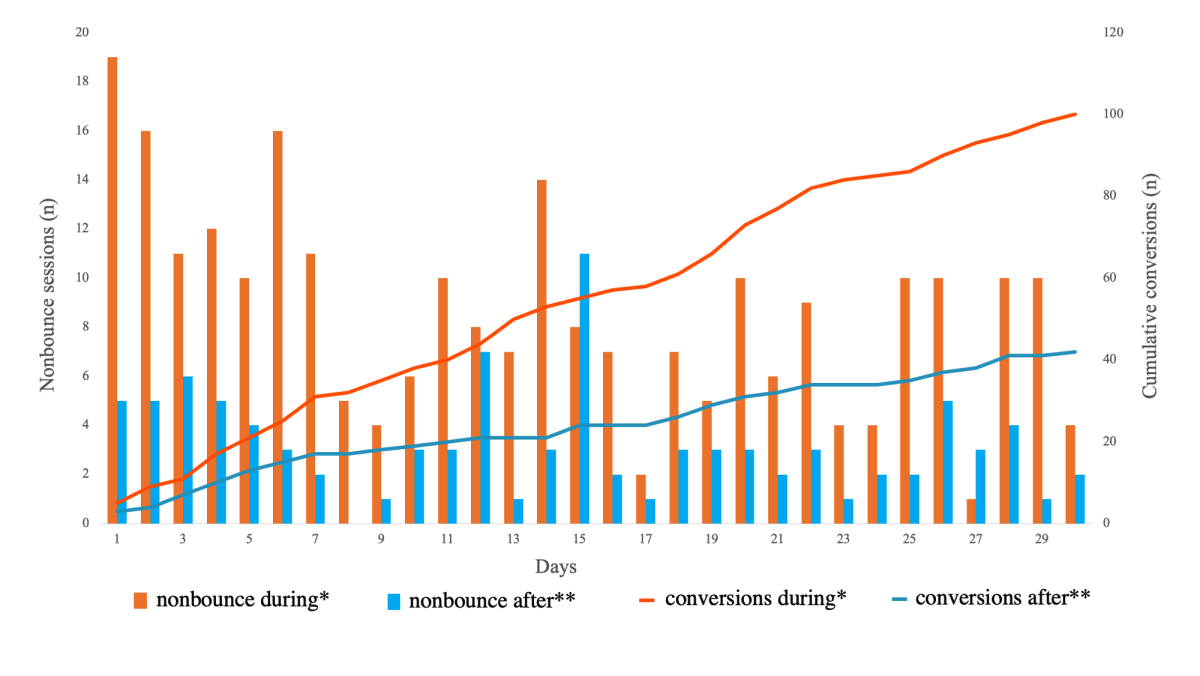
Number of nonbounce users and cumulative conversions during and after the ad campaign.*The 30-day period during the ad campaign (January 26, 2018 to February 24, 2018). **The 30-day period after the ad campaign (March 23, 2018 to April 21, 2018).

The website attracted 1311 users during the 30-day ad campaign and 241 users in the 30-day period selected after the ad campaign. The average daily user count was higher (43.7 vs 8.0; *P*<.001) and the return visitor rate was lower (17.7%, 232/1311, vs 27.8%, 67/241; *P*<.001) during the ad campaign (Figure 5). The rate of nonbounce visits was lower during the ad campaign (19.5%, 256/1311, vs 39.8%, 96/241; *P<*.001). Similarly, the session duration was shorter (42, SD 27, vs 102, SD 118 seconds; *P*=.009) and the average number of pages viewed per session was lower (1.4, SD 0.2, vs 2.1, SD 0.7; *P*<.001) during the ad campaign. The number of 30-day conversions was higher during the ad campaign (100 vs 47; *P<*.001), with an associated lower conversion rate based on the number of sessions (7.1%, 100/1401, vs 15.9%, 42/264; *P*<.001; Figure 6).

Detailed website behavior was available for 1400 sessions during the ad campaign. Advertising via display ads and paid searches accounted for 59.9% (839/1400) of the website sessions during the 30-day periods. The behavior of users visiting the website because of advertising differed from that of other visitors: The bounce rate was higher, pages viewed was lower, and visit duration was shorter when users were directed to the site via advertising (Table 3). In addition, 9 of the 839 (1.1%) sessions with one or more conversions were prompted by the Headstrong website ads as compared to 51 of the 561 (9.1%) sessions unprompted by an advertisement during the same 30-day period (*P<*.001).

#### Acquisition

**Figure 7:**
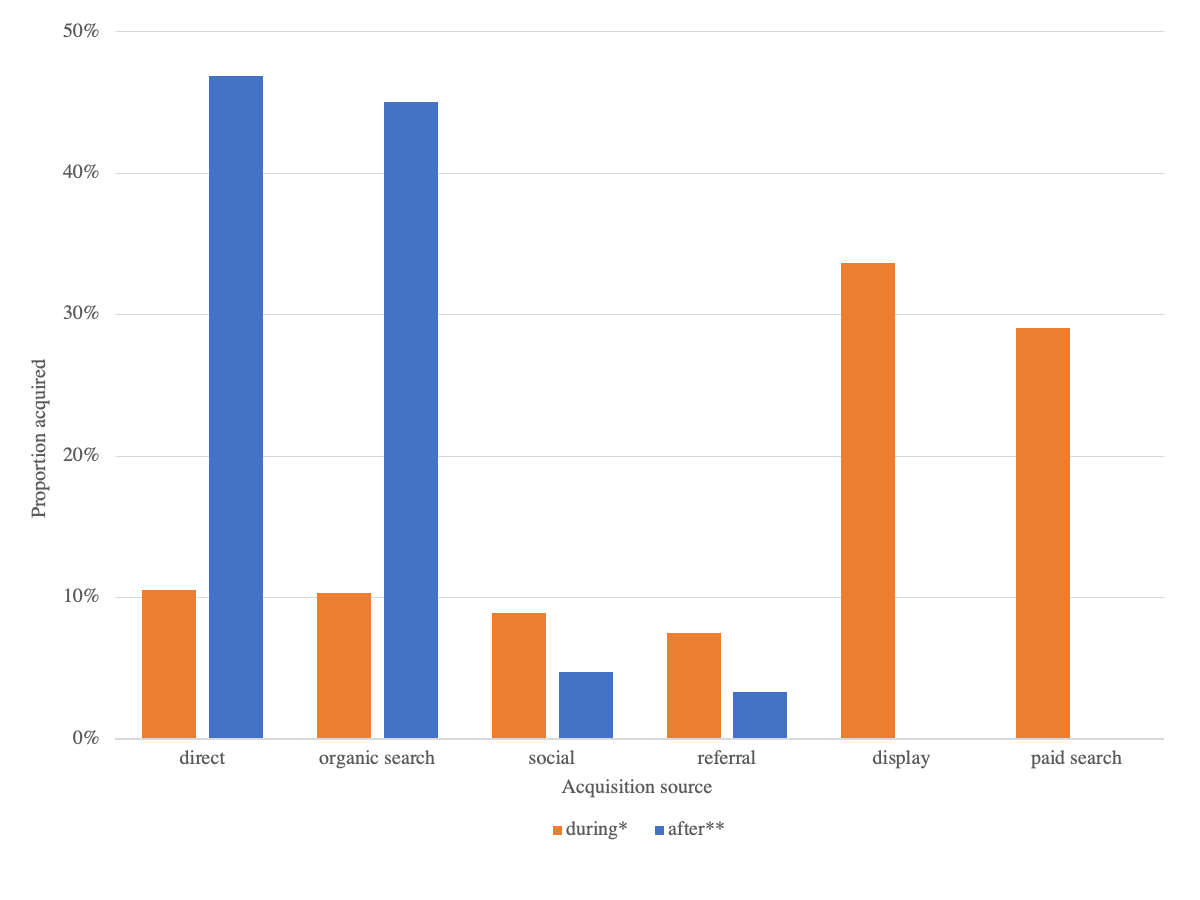
Website acquisition sources during (n=1203) and after (n=211) the Headstrong website ad campaign.*The 30-day period during the ad campaign (January 26, 2018 to February 24, 2018). **The 30-day period after the ad campaign (March 23, 2018 to April 21, 2018).

Website acquisition data were available for 91.8% (1203/1311) of users during and 87.6% (211/241) of users after the 30-day campaign periods. Paid and display ads accounted for 62.7% (754/1202) of site traffic during the ad campaign (Figure 7). The combined number of users from direct, organic search, social media, and referral sources during the 30-day period of the ad campaign was greater than that in the selected 30-day period after the ad campaign (448 users vs 211 users, *P*<.001).

**Table 3:** Website user behavior based on acquisition source during the 30-day ad campaign period.

Source		Sessions, n	Bounce rate, %	Pages per session, n	Mean session duration (seconds)	Sessions with conversions, n (%)^a^
**Google Ads**						
	Display	437	85.4	1.2	13	6 (1.4)
	Paid Search	402	92.3	1.1	18	3 (0.8)
**Other**						
	Direct	164	57.9	2.0	120	21 (12.8)
	Organic search	177	67.8	1.7	70	18 (10.2)
	Social	126	66.7	1.6	52	11 (8.7)
	Referral	94	93.6	1.2	54	1 (1.1)

^a^Sessions can have one or more conversions. Percentages indicate the number of sessions with conversions divided by the total number of sessions.

**Table 4:** Devices used to access the Headstrong website during and after the Google Ad campaign.

Devices		Bounce rate, %	Pages or session, n	Average session duration, seconds	Conversions, n
**During the ad campaign (30 days, n=1195)**					
	Mobile	87.3	1.2	19	18
	Desktop	57.8	2.0	112	36
**After the ad campaign (30 days, n=206)**					
	Mobile	78.2	1.7	74	6
	Desktop	51.8	2.4	129	23

#### Devices

Data on which device was utilized by website users when accessing the Headstrong website were available for 91.2% of all users (1195/1311) during the 30-day period of the ad campaign and 85.5% of all users (206/241) in the selected 30-day period after the ad campaign. Mobile phones represented the majority of devices used during the 30-day period of the ad campaign (74.0%, 884/1195), followed by desktop computers (19.9%, 238/1195) and tablets (6.1%, 73/1195). After the ad campaign, device use differed substantively (*P*<.001): Desktop computers were used most often (51.5%, 106/206), followed by mobile phones (40.8%, 84/206) and tablets (7.8%, 16/206).

During and after the ad campaign, mobile phone users had a higher bounce rate, fewer pages per session, shorter average session duration, and lower number of conversions than desktop computer users. In addition, the number of conversions with mobile phone users was half that of desktop users, although mobile phone users accounted for the majority of users during the ad campaign (Table 4). No differences in device use were observed on the basis of gender (data not shown).

**Table 5:** Analytics of ad groups during the 30-day Google Ads campaign.

Google Ad group	Clicks, n	Cost per click, Can $	Bounce rate, %	Conversions, n (%)
Ad Group #1	717	0.53	93.2	1 (0.2)
Ad Group #1_Depression	205	0.23	84.5	5 (3.0)
Ad Group #1_Bipolar	91	0.25	79.6	1 (1.1)
Ad Group #1_Disorder	84	0.23	84.5	0 (0.0)
Ad Group #1_Mental	32	0.26	89.7	0 (0.0)
Ad Group #1_Mental Illnesses	23	0.28	90.9	0 (0.0)
Ad Group #1_Depression_Causes Depression	11	0.22	100.0	0 (0.0)
Ad Group #1_Smoking	4	0.80	100.0	0 (0.0)
Ad Group #1_Mental Health	2	0.62	33.3	2 (66.7)
Ad Group #1_Mental Disorders	1	0.79	100.0	0 (0.0)
Total	1171	0.42	88.7	9 (1.1)

#### Performance of Google Ads Groups

Ad group performances were relatively low overall, with high bounce rates and few conversions. The most general ad group, “Ad Group #1,” generated the highest number of clicks at 717; however, only one conversion was noted with this group (Table 5). Moreover, for this ad group, “Nova Scotia” was included as a part of the search string in 91.6% (657/717) of clicks and target content terms were included in 6.3% (45/717) of clicks. The search term “men” was included in only 2.1% (15/717) of the search terms but demonstrated the best engagement, with a bounce rate of 80% and the highest number of pages per session (1.4) within this ad group. The more specific “Ad Group #1_Depression” demonstrated the best performance overall, with 205 clicks, lower cost per click compared to “Ad Group #1,” an intermediate bounce rate, and a good conversion rate of 3%.

#### Cost Analysis

The cost of the 30-day period during the campaign was $492.20, with an average cost of $16.40 per day. The click-through rate was 0.1%, with an average cost per click of $0.42 and a conversion rate of 1.1%. A total of 9 sessions had one or more conversions resulting from a Google Ads campaign advertisement. The cost per session with conversion was $54.69. Assuming that the number of conversions per session was 1.67 (100 conversions in 60 sessions with conversions) was the same irrespective of whether the site user was acquired by a paid ad, we estimate that 15 conversions resulted from the ad campaign. This corresponds to $32.81 per conversion.

## Discussion

### Principal Findings

A Google Ads campaign substantially increased the number of visits to the Headstrong men’s mental health website. Although there were more user visits because of the ad campaign, the analytics data demonstrated a substantial reduction in user engagement. Higher bounce and lower return visitor rates, lower visit duration, and fewer page views suggested that the ad campaign attracted visits from individuals who were not interested in Headstrong’s purpose or web content. Targeting men, in general, through an online ad campaign that encouraged them to find support and resources for depression, anxiety, insomnia, and other mental health issues was not successful. This could be, in large part, due to the use of nonspecific keywords along with content-specific keywords for the ad campaign. Our results showed that this approach was not efficient, despite a relatively high prevalence of depression, anxiety disorders, insomnia, and tobacco and alcohol use problems in men. It is also possible that the nonspecific group of online users who saw and clicked on the Google Ad, thus using a portion of the daily spending limit of $15, prevented the ad from being viewed by someone more specifically targeted on the same day.

### Overview

A primary aim of the Headstrong initiative was to help men access self-help resources for anxiety, depression, insomnia, and tobacco and alcohol use problems and to directly seek help for thoughts of suicide. For each mental health and addictions issue, a limited set of vetted and recommended resources were described succinctly with video overviews. Outbound clicks (conversions) to these resources indicated that users were engaged and potentially interested in accessing the resource. There were more conversions during the paid ad campaign, but a closer examination of user behavior per session demonstrated that the ads had little impact on conversions. Only 9 of the 60 sessions (15%) with conversions during the specified 30-day period of the ad campaign were from users who accessed the Headstrong website via an ad.

Most conversions were from sessions initiated independent of the paid ads via a variety of mechanisms, including the in-pharmacy Headstrong initiative. The number of conversions during the ad campaign was higher than that after the ad campaign due to a higher number of visitors who sought out the Headstrong website intentionally. This may, in part, be a remnant of the increased traffic that followed the call-in public radio show that brought attention to the website and more activity within participating pharmacies in the early stages of the Headstrong initiative.

The majority of responses to the ad campaign were from mobile device users, suggesting that they were more plentiful or more sensitive to the ads than desktop users. The higher proportion of mobile device users during the ad campaign is consistent with the findings of Birnbaum (2017), who reported a higher responsiveness to ads from mobile device users than from desktop users [16]. Independent of the ad campaign, we observed lower website engagement by mobile device users than by desktop users. The reasons for higher responsiveness and lower engagement from the mobile users is unclear. We believe this may be due to the volume of online searches using mobile devices as compared to desktop devices and differences in the website appearance among devices. Our observations reinforce the importance of developing engaging, mobile-friendly websites. Further work is needed to determine how to best improve engagement of mobile device users in response to the global trend of increasing mobile device use [25].

A common method to characterize the cost of a Google Ads campaign is to report the cost per conversion of a webpage. For the Headstrong webpage, the desired outcome was for users to click on one of the several outbound links to the recommended mental health resources. We estimate the investment per conversion to be $33 or $55 per session. The cost data for other Google Ads health-related campaigns are considerably varied, with cost per desired outcome ranging between US $6.70 [26] to Aus $495 [15]. Although the cost of our ad campaign was on the higher end, it was favorable as compared to other advertisement campaigns [11,13,15-17,20,22,26,27]. However, for an initiative that does not generate revenue, the cost of the ad campaign as currently designed is unsustainable.

Our experience with this Google Ads campaign highlights the importance of regular evaluation and modification of a campaign’s keywords to optimize the impact of the investment. Keywords that lead to a high volume of clicks but undesired behaviors (ie, high bounce rates, brief visit durations, and few conversions) will result in the inefficient use of a campaign’s daily budget and thereby limit advertising to more appropriate, targeted online users. However, it is important to recognize that focusing the ad campaign on a narrower target user changes the purpose of the campaign. For our campaign, narrowing the keywords would increase our ability to support men who are actively searching for information and resources on mental health and addiction. However, it would reduce the ability to reach men who may benefit from such information and resources even though they were not actively looking for such information [11].

### Limitations

A substantial proportion of the demographic details of website users were unavailable, and users were able to prevent tracking of their general location, age, and gender. This limited our ability to determine differences in user behavior based on these variables. The desired behavior of users of the Headstrong website was the use of the recommended resources. Our proxy measure for identifying this behavior is determining conversions based on the use of outbound links to the recommended resources. We were unable to determine whether the user actually benefited from the resource.

### Conclusions

A Google Ads campaign designed to direct men to Headstrong website, which presents a curated collection of print and online recommended resources for depression, anxiety, insomnia, tobacco and alcohol use problems, and suicide risk, increased the number of user visits by more than five-fold. People using mobile devices were most responsive to the campaign. Engagement by users responding to the ad campaign was substantially lower than that by users who visited the website via other acquisition methods. The use of nonspecific keywords accounted for most visits but may have failed to attract men interested in accessing resources focused on mental health and specific substance use problems. Narrowing the keywords may result in more efficient use of ad campaign funds with greater user engagement.
